# Small Airway Dysfunction in Rheumatoid Arthritis-Associated Interstitial Lung Disease: A Single-Center Retrospective Observational Study

**DOI:** 10.3390/jcm15156035

**Published:** 2026-08-03

**Authors:** Xiaoyan Wang, Yu Xu

**Affiliations:** Department of Pulmonary and Critical Care Medicine, Beijing Jishuitan Hospital, Capital Medical University, Beijing 100035, China; pkuph_xuyu@163.com

**Keywords:** rheumatoid arthritis, interstitial lung disease, small airway dysfunction, impulse oscillometry, pulmonary function, disease severity

## Abstract

**Background:** Rheumatoid arthritis-associated interstitial lung disease (RA-ILD) is an important extra-articular manifestation of rheumatoid arthritis and is associated with impaired pulmonary function and poor clinical outcomes. Although interstitial abnormalities in RA-ILD have been widely studied, airway involvement, particularly small airway dysfunction, has received less attention. This study aimed to evaluate small airway function and impulse oscillometry parameters in patients with RA-ILD and to explore their potential associations with disease severity. **Methods:** This is a hospital-based cross-sectional comparative observational study using retrospectively collected clinical data. The patients with RA-ILD included in this study were treated at Beijing Jishuitan Hospital, Capital Medical University, between January 2021 and December 2025. Age- and sex-matched healthy individuals without pulmonary disease were included as controls. Clinical data, high-resolution computed tomography (HRCT) findings, pulmonary function parameters, and impulse oscillometry indices were collected. Small airway function was assessed using MEF25–75%, MEF50%, and MEF25%. Disease severity was evaluated using HRCT visual scores, the gender–age–physiology (GAP) score, and the composite physiologic index (CPI). Subgroup analyses were performed according to the presence of small airway dysfunction, HRCT subtype, and history of RA-related joint surgery. Correlations between airway function parameters and disease severity indices were analyzed. Multiple linear regression analyses were performed. **Results:** A total of 255 patients with RA-ILD were included, including 85 men (33.3%), with a mean age of 67.53 years. Compared with controls, patients with RA-ILD had significantly lower BMI, FVC%, TLC%, and DLCO% (all *p* < 0.05). Small airway function parameters, including MEF25–75%, MEF50%, and MEF25%, were significantly reduced in the RA-ILD group. Impulse oscillometry showed significantly higher Z5, R5–R20, R5/R20, and Fres, and significantly lower X5 in patients with RA-ILD compared with controls. Patients with RA-ILD and small airway dysfunction had lower FVC% than those without small airway dysfunction (87.7% vs. 95.46%, *p* = 0.015), and their GAP scores tended to be higher, although the difference did not reach statistical significance (2.59 vs. 1.79, *p* = 0.055). Among different ILD subtypes, only MEF50% differed significantly among the UIP, NSIP, and other-pattern groups. No significant differences in airway function or disease severity were observed between patients with and without a history of RA-related joint surgery. Correlation analysis showed that MEF25–75%, MEF50%, and MEF25% were negatively correlated with GAP score and positively correlated with FVC%. MEF25–75% and MEF50% were also positively correlated with DLCO%. **Conclusions:** In this study, patients with RA-ILD showed evidence of small airway dysfunction, with altered impulse oscillometry parameters suggesting increased airway resistance and reduced airway compliance. Small airway function parameters were associated with FVC% and GAP score, indicating that small airway dysfunction may be related to disease severity in RA-ILD.

## 1. Introduction

Rheumatoid arthritis (RA) is a systemic autoimmune disease characterized primarily by symmetric polyarticular synovitis, with a global prevalence of 0.46% [1]. Pulmonary involvement is a common extra-articular manifestation. Rheumatoid arthritis-associated interstitial lung disease (RA-ILD), an important pulmonary complication, has been reported in approximately 10–50% of patients with RA [2]. Compared with RA patients without ILD (RA–non-ILD), patients with RA-ILD have a 2–10-fold higher mortality risk, and the median survival after diagnosis is less than 3 years [3].

The airway mucosa may represent an initial site of RA-related immune activation [4,5]. CT studies have shown airway abnormalities in up to 61% of RA patients [6,7], mainly including bronchiectasis, bronchiolitis, and airway wall thickening, with airflow limitation observed in 20.7% of patients. Quantitative HRCT analyses suggest that airway wall thickening, emphysema, and related imaging metrics are associated with respiratory symptom severity in RA.

However, airway functional abnormalities in RA-ILD remain insufficiently characterized, and their relationship with disease severity is not well established. Evidence from large, targeted clinical studies is still limited. Therefore, systematic evaluation of airway function in RA-ILD may help improve understanding of its pathophysiology and provide supplementary information for clinical assessment. The primary objective of this study was to compare small airway function parameters between patients with RA-ILD and healthy controls, in order to characterize potential differences in small airway function. The secondary objective was to explore associations between small airway function parameters and indices of RA-ILD severity, thereby providing preliminary descriptive evidence regarding small airway functional abnormalities in RA-ILD.

## 2. Methods

This is a hospital-based cross-sectional comparative observational study using retrospectively collected clinical data.

### 2.1. Study Population

**Figure 1:**
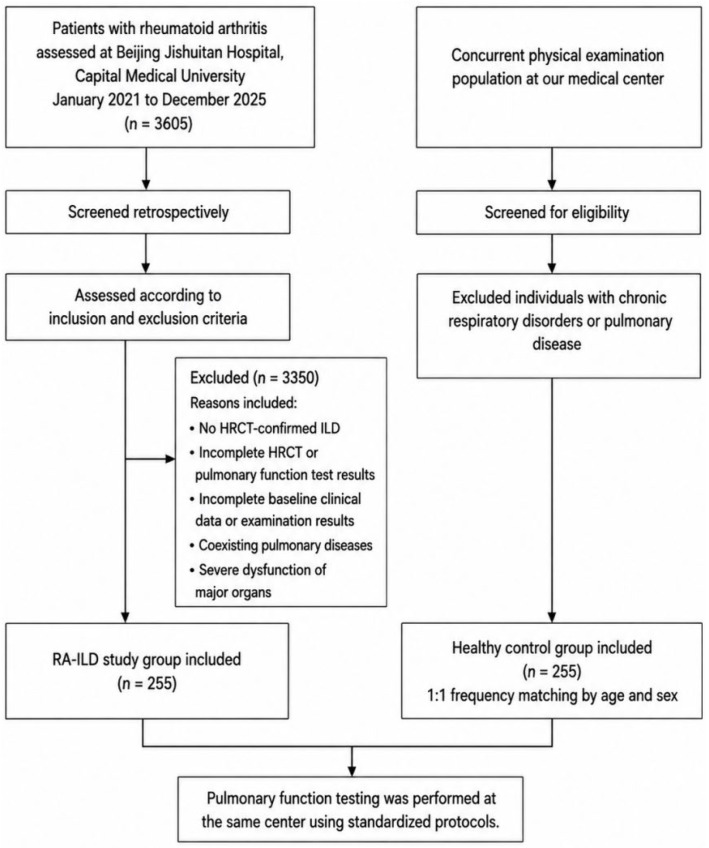
Flow diagram of participant selection.

Among 3605 patients with rheumatoid arthritis who visited Beijing Jishuitan Hospital, Capital Medical University, between January 2021 and December 2025, patients diagnosed with RA-ILD were selected retrospectively as the study group (Figure 1).

Healthy controls were selected from concurrent physical examination populations in our medical center at a matching ratio of 1:1, strictly matched by gender and age. Individuals with chronic respiratory disorders were excluded from the control group.

Ethical review and approval were waived for this study due to its retrospective design and the absence of identifiable patient information that could compromise individual privacy.

### 2.2. Inclusion Criteria

Patients were included if they met the following criteria:

1. Fulfillment of the 2010 American College of Rheumatology/European League Against Rheumatism classification criteria for RA [8];
2. Diagnosis of ILD confirmed by chest HRCT and completion of comprehensive pulmonary function testing;
3. Availability of complete clinical baseline data and examination results.

### 2.3. Exclusion Criteria

Patients were excluded if they met any of the following criteria:

1. Coexisting pulmonary infection, lung cancer, bronchiectasis, asthma, chronic obstructive pulmonary disease, or other pulmonary diseases;
2. Incomplete chest HRCT or pulmonary function test results;
3. Severe dysfunction of major organs, including the heart, liver, or kidneys.

### 2.4. Baseline Data Collection

General demographic and clinical data were collected uniformly for all participants, including sex, age, smoking history, body mass index, comorbidities such as hypertension, diabetes mellitus, coronary heart disease, and cerebrovascular disease, as well as RA-related clinical data.

### 2.5. HRCT Examination and Disease Assessment

HRCT images were independently reviewed by two experienced radiologists who were blinded to patients’ clinical diagnosis, pulmonary function results, and disease severity assessments, as well as to each other’s interpretations. Disagreements were resolved by discussion and consensus. ILD was diagnosed and classified according to HRCT findings into usual interstitial pneumonia (UIP), nonspecific interstitial pneumonia (NSIP), or other patterns. ILD severity was assessed using a visual CT scoring system. The gender–age–physiology (GAP) score and the composite physiologic index (CPI) were also calculated to comprehensively evaluate disease severity. The GAP score and CPI were calculated as supplementary physiologic indices to describe disease severity. As both models were originally developed and validated in idiopathic pulmonary fibrosis, their use in RA-ILD was exploratory.

For the visual CT score of ILD severity [9], each lung lobe was scored from 0 to 5 according to the percentage of interstitial abnormalities. The total fibrosis score ranged from 0 to 25, and severity was classified as mild (0–8), moderate (9–16), or severe (17–25).

The GAP score was calculated according to the scoring and staging method proposed by Ley et al. in 2012 [10]. Sex, age, forced vital capacity percent predicted, and diffusing capacity of the lung for carbon monoxide percent predicted were scored, and the total score was used as the GAP score. Patients were classified into three stages according to the total GAP score: stage I, 0–3 points; stage II, 4–5 points; and stage III, 6–8 points.

The CPI was used to describe overall pulmonary function status in patients with IPF [11]. Three parameters were included in the formula: diffusing capacity of the lung for carbon monoxide percent predicted (DLCO%), forced vital capacity percent predicted (FVC%), and forced expiratory volume in 1 s percent predicted (FEV1%). Patients with IPF were divided into two groups according to the CPI [12]: low CPI group, CPI ≤ 41, and high CPI group, CPI > 41.

The CPI was calculated as follows:

CPI = 91.0 − (0.65 × DLCO%pred) − (0.53 × FVC%pred) + (0.34 × FEV1%pred).

### 2.6. Pulmonary Function Testing

Comprehensive pulmonary function testing was performed using standardized pulmonary function equipment [13]. The following parameters were recorded: ventilatory function indices, including FEV1%, FVC%, and FEV1/FVC ratio; small airway function indices, including maximum expiratory flow between 75% and 25% of FVC (MEF25–75%), maximum expiratory flow at 50% of FVC (MEF50%), and maximum expiratory flow at 25% of FVC (MEF25%); lung volume index, including total lung capacity percent predicted (TLC%); diffusing capacity index, DLCO%; and impulse oscillometry indices, including respiratory impedance at 5 Hz (Z5), the difference between airway resistance at 5 Hz and 20 Hz (R5–R20), R5/R20, reactance at 5 Hz (X5), and resonant frequency (Fres).

### 2.7. Subgroup Analyses

Small airway dysfunction was defined as at least two of the following three indices being less than 65% of the predicted value: MEF25–75%, MEF50%, and MEF25% [14]. According to the presence or absence of small airway dysfunction, patients with RA-ILD were divided into a small airway dysfunction group and a non-small airway dysfunction group, and disease severity was compared between the two groups.

According to HRCT patterns of RA-ILD, patients were further divided into the UIP group, NSIP group, and other-pattern group. Small airway function indices were compared among these groups to explore the potential impact of ILD subtype on small airway function.

According to whether patients had undergone RA-related joint surgery, RA-ILD patients were divided into a surgery group and a non-surgery group. In this study, a history of RA-related joint surgery was considered to indicate more severe joint involvement. Small airway function was compared between the two groups to assess whether it differed according to the severity of joint disease.

### 2.8. Statistical Analysis

Data were analyzed using SPSS version 26.0. Continuous variables are expressed as mean ± standard deviation. Between-group comparisons were performed using the *t* test, analysis of variance, or nonparametric tests, as appropriate. Categorical variables are expressed as rates or percentages, and between-group comparisons were performed using the χ^2^ test. Baseline characteristics were compared between groups. Pulmonary function, particularly small airway function and impulse oscillometry parameters, was compared between groups. Pearson or Spearman correlation analysis was used to evaluate associations between airway function parameters and disease severity. All pairwise correlation analyses are exploratory descriptive analyses without multiple comparison adjustment. Multiple linear regression analyses were performed to evaluate the associations between small airway function parameters and disease severity. A two-sided *p* value < 0.05 was considered statistically significant.

This retrospective observational study was reported in accordance with the Strengthening the Reporting of Observational Studies in Epidemiology (STROBE) statement.

## 3. Results

A total of 255 patients with RA-ILD were included in the study, including 85 men (33.3%). The mean age was 67.53 years, and 25.1% of the patients had a history of smoking. BMI, FVC%, TLC%, and DLCO% were significantly lower in the RA-ILD group than in the control group, with statistically significant differences (*p* < 0.05). Details are shown in Table 1.

### 3.1. Comparison of Small Airway Function Parameters Between the RA-ILD Group and the Control Group

**Figure 2:**
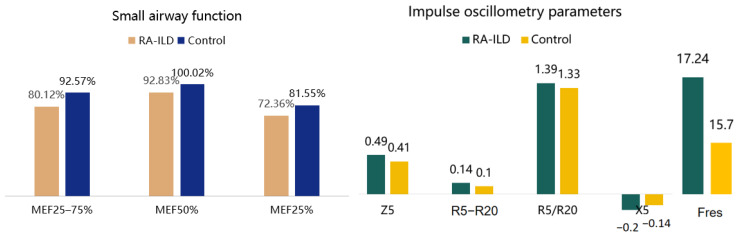
Comparison of small airway function and impulse oscillometry parameters between the RA-ILD group and the control group.

Small airway function parameters were significantly lower in the RA-ILD group than in the control group, and the between-group differences were statistically significant (*p* < 0.05) (Figure 2). Impulse oscillometry showed that Z5, R5–R20, R5/R20, and Fres were significantly higher in the RA-ILD group than in the control group, whereas X5 was significantly lower. All between-group differences were statistically significant (*p* < 0.05) (Figure 2).

The predicted values were derived from the mean values of subjects matched for age, sex, height and weight. Measured small airway function parameters were compared with corresponding predicted values and are expressed as the percentage of predicted values. Comparison of the percentage of predicted small airway function parameters between the RA-ILD group and the control group revealed statistically significant intergroup differences (*p* < 0.05, Table 2).

### 3.2. Subgroup Analysis of Patients with RA-ILD

Patients with RA-ILD were divided into a small airway dysfunction group and a non-small airway dysfunction group according to the presence or absence of small airway dysfunction. FVC% was significantly lower in the small airway dysfunction group than in the non-small airway dysfunction group (87.7% vs. 95.46%, *p* = 0.015). The GAP score was higher in the small airway dysfunction group than in the non-small airway dysfunction group (2.59 vs. 1.79), although the between-group difference did not reach statistical significance (*p* = 0.055).

**Figure 3:**
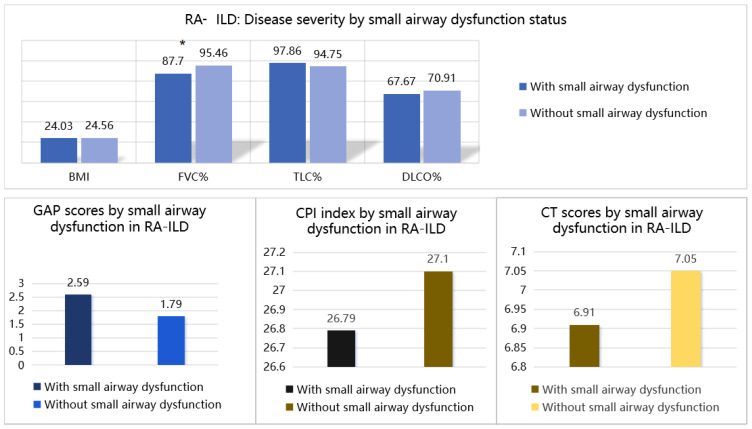
Comparison of disease severity between RA-ILD patients with and without small airway dysfunction; *: *p* < 0.05.

There were no statistically significant differences between the two groups in BMI, TLC%, DLCO%, CPI, or CT extent score (all *p* > 0.05) (Figure 3).

Patients with RA-ILD were classified into the UIP, NSIP, and other-pattern groups according to ILD subtype. Comparisons among the three groups showed a statistically significant difference in MEF50% across ILD subtypes (117.24, 90.08, and 74.05, respectively; *p* = 0.034).

**Figure 4:**
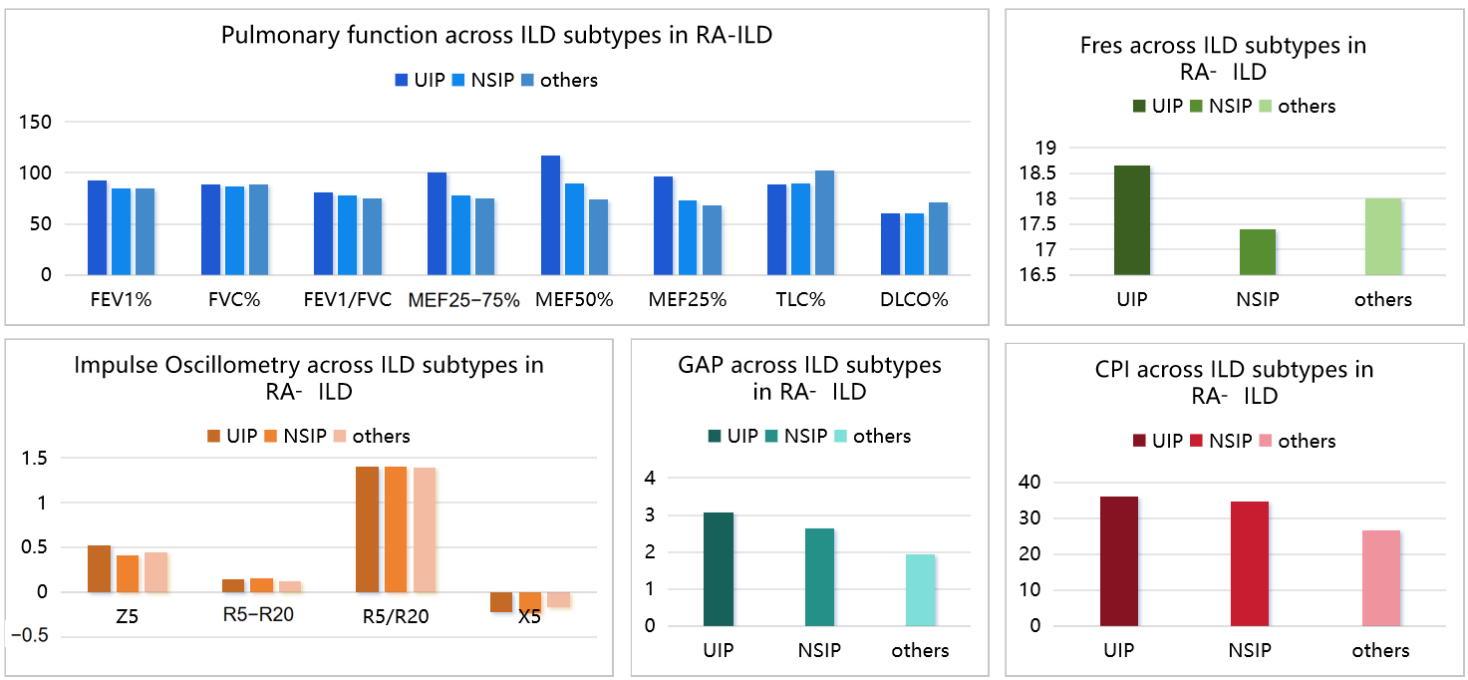
Comparison of pulmonary function among RA-ILD patients with different ILD subtypes.

No statistically significant differences were observed among the subtype groups in ventilatory function parameters, including FEV1%, FVC%, and FEV1/FVC; small airway function parameters, including MEF25–75% and MEF25%; impulse oscillometry parameters, including Z5 and R5–R20; or TLC%, RV/TLC, DLCO%, GAP score, and CPI (all *p* > 0.05) (Figure 4).

Patients with RA-ILD were divided into a surgery group and a non-surgery group according to whether they had undergone RA-related joint surgery. No statistically significant differences were observed between the two groups in ventilatory function parameters, small airway function parameters, impulse oscillometry parameters, volumetric parameters, GAP score, or CPI (all *p* > 0.05).

**Figure 5:**
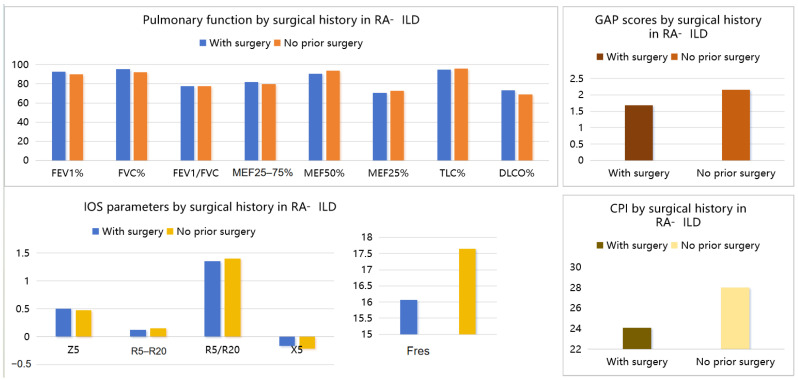
Comparison of disease severity between RA-ILD patients with and without a history of RA-related joint surgery.

It suggests that a history of RA-related joint surgery was not clearly associated with differences in pulmonary function or disease severity-related indices (Figure 5).

### 3.3. Potential Correlations Between Small Airway Function Parameters and Disease Severity

**Figure 6:**
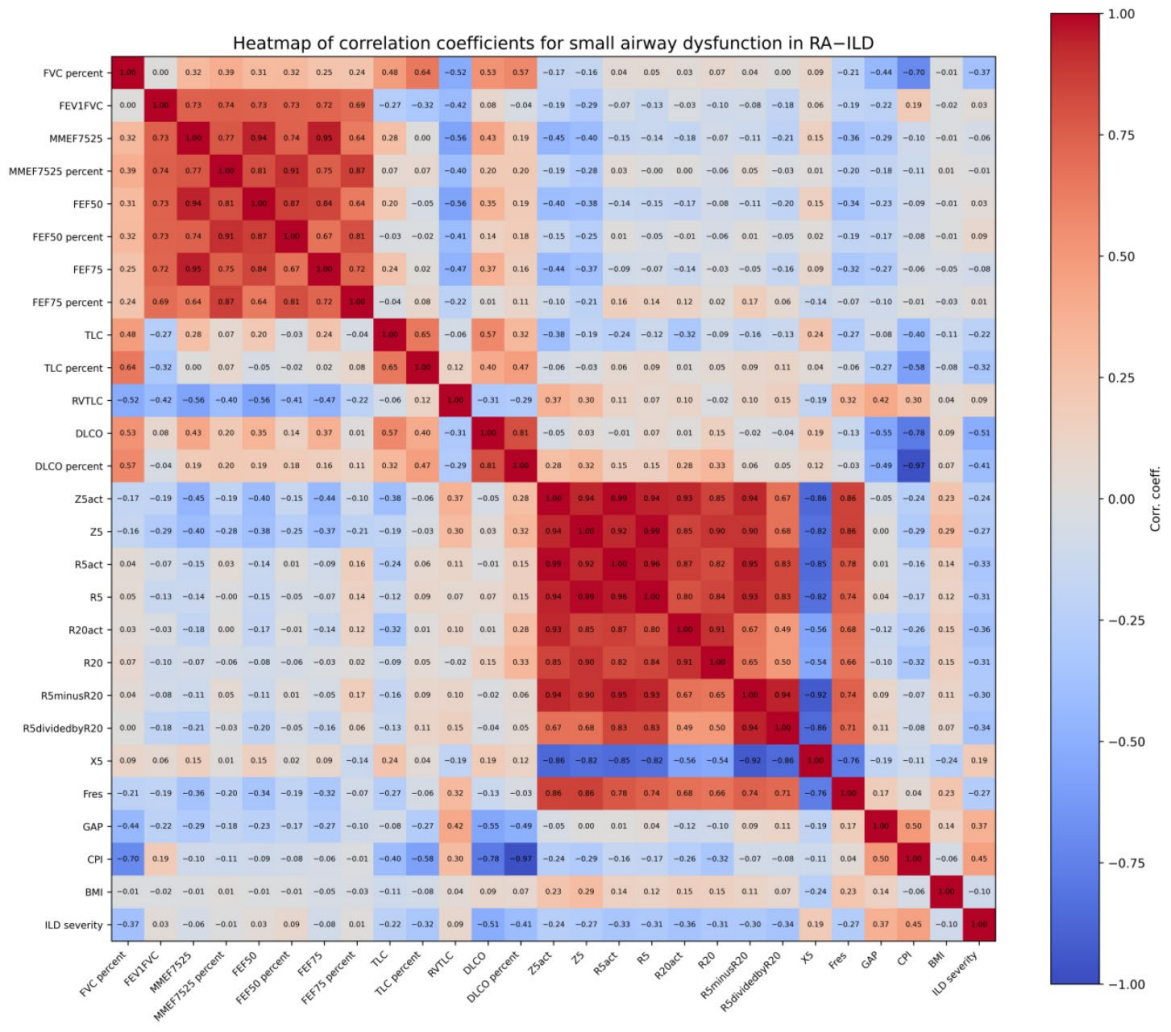
Potential correlations between small airway function parameters and disease severity. The heatmap shows correlations among small airway function parameters, impulse oscillometry indices, pulmonary function parameters, and disease severity-related variables. Red indicates positive correlations, blue indicates negative correlations, and color intensity reflects the magnitude of the correlation coefficient.

Pearson correlation analysis showed that small airway function parameters were significantly associated with disease severity and key pulmonary function parameters in patients with RA-ILD (Supplementary Materials). MEF25–75%, MEF50%, and MEF25% were all significantly negatively correlated with the GAP score (*p* < 0.05). MEF25–75%, MEF50%, and MEF25% were all significantly positively correlated with FVC% (*p* < 0.01). In addition, MEF25–75% and MEF50% were significantly positively correlated with DLCO% (*p* < 0.05). Among impulse oscillometry parameters, Z5 was significantly positively correlated with DLCO% (*p* < 0.05) (Figure 6).

Multiple linear regression analyses were conducted. Disease severity-related indicators (GAP stage, FVC% predicted and DLCO% predicted) were set as dependent variables, and small airway function parameters were treated as independent variables, with sex, age, height, weight and smoking status adjusted as confounding factors. After adjusting for age, sex, height, weight and smoking status, small airway function indices (MEF25–75%pred, MEF50%pred, MEF25%pred) were independently associated with disease severity indicators (GAP stage, FVC%pred, DLCO%pred) (Table 3).

## 4. Discussion

In this study, analysis of clinical data from a relatively large cohort showed that patients with RA-ILD had evidence of airway functional abnormalities, mainly involving small airway function. Small airway function parameters showed potential associations with selected indices of RA-ILD disease severity. These findings suggest that small airway function parameters may provide descriptive information regarding airway involvement in RA-ILD. However, because this study was retrospective and cross-sectional, these associations should not be interpreted as evidence of independent clinical utility, prognostic value, or monitoring capability. Whether small airway function parameters or IOS indices provide additional information beyond conventional pulmonary function tests and HRCT requires further validation in prospective studies.

Pulmonary involvement in RA may affect multiple anatomical structures, including the interstitium, airways, and pulmonary vasculature, and may manifest as interstitial lung disease, bronchiectasis, and other pulmonary abnormalities [15]. Previous studies have mainly focused on the interstitial features, prognosis, and treatment strategies of RA-ILD, whereas changes in airway function have received comparatively less attention. In the present study, patients with RA-ILD showed reductions not only in FVC%, TLC%, and DLCO%, reflecting impaired ventilatory and diffusing capacity, but also in small airway function parameters, including MEF25–75%, MEF50%, and MEF25%. Patients with RA-ILD and small airway dysfunction had lower FVC%, and their GAP scores tended to be higher. Correlation analysis further showed that small airway function parameters, including MEF25–75%, MEF50%, and MEF25%, were negatively correlated with GAP score and positively correlated with FVC% and DLCO%. These findings suggest that small airway dysfunction may be associated with impaired ventilatory function and disease severity in patients with RA-ILD. Small airway function parameters may reflect certain aspects of physiological impairment in RA-ILD, but their role in severity assessment requires further validation. Whether these indices provide information beyond conventional parameters such as FVC% and DLCO% remains uncertain and requires further prospective validation.

Comparative analysis among different ILD subtypes showed that only MEF50% differed significantly among the UIP, NSIP, and other-pattern groups, whereas no significant differences were observed in other airway function parameters, impulse oscillometry parameters, or disease severity indices. These findings suggest that airway functional abnormalities in RA-ILD may not be strongly associated with the pathological subtype of ILD. Airway involvement in RA-ILD may represent a common manifestation of systemic autoimmune injury in RA rather than a secondary change specific to a particular ILD subtype. This also suggests that airway involvement and interstitial lesions may represent two relatively independent pathological processes in RA-related pulmonary disease. Small airway spirometric indices should be interpreted cautiously in RA-ILD. HRCT interpretation and visual scoring may be influenced by interobserver variability. Although disagreements between the two radiologists were resolved by consensus, individual readings were not recorded separately; therefore, interobserver agreement statistics could not be calculated. This may have introduced some uncertainty in HRCT subtype classification and visual severity scoring.

Previous studies have suggested that the bronchial mucosa may be involved in RA-related immune activation, including airway inflammation, epithelial citrullination, and local anti-citrullinated protein antibody responses [16,17,18,19,20,21,22,23]. These findings provide a possible background for airway involvement in RA, although the present study did not directly investigate immunopathological mechanisms. Small airway dysfunction has also been reported in other connective tissue disease-associated lung diseases, including systemic sclerosis and Sjögren disease [24,25].

The small airways are delicate pulmonary structures with narrow lumens, thin walls, and no cartilaginous support. They may therefore be particularly susceptible to inflammatory and immune-mediated injury. Early small airway lesions often lack obvious clinical symptoms, and conventional ventilatory parameters may remain normal. Assessment of small airway function parameters and impulse oscillometry may help describe airway functional abnormalities. In the present study, compared with controls, patients with RA-ILD had increased Z5, R5–R20, and Fres, and decreased X5. Impulse oscillometry is a noninvasive method that applies forced oscillatory signals during quiet tidal breathing to assess airway resistance and reactance. R5–R20 is commonly used as an estimate of small airway resistance. An increase in R5–R20 may indicate peripheral small airway dysfunction and may occur before abnormalities in conventional pulmonary function parameters, such as FEV1/FVC and MEF. Thus, it may be related to small airway impairment, although its role as a screening marker in RA-ILD remains to be established. Previous studies have suggested that oscillometry may reflect functional changes in the small or peripheral airways; however, its incremental value over conventional pulmonary function testing in RA-ILD remains uncertain [26].

Low-frequency oscillatory signals can reach the peripheral airways; therefore, X5 may reflect the overall elastic properties of the airway system, particularly those of the peripheral small airways. Yamamoto et al. reported that X5, Fres, and low-frequency reactance area (ALX/AX) were positively correlated with HRCT fibrosis scores, whereas vital capacity and FVC were negatively correlated with oscillometry parameters and HRCT fibrosis scores [27]. Gogali et al. found that 27% of patients with early IPF had abnormal R5–R20, which was associated with MEF25–75% < 60% predicted, suggesting its potential value as an early indicator of small airway dysfunction [28]. Wu et al. also reported that end-inspiratory reactance and inspiratory X5 were strongly correlated with FVC, further supporting the potential relationship between oscillometry parameters, restrictive pulmonary impairment, and small airway mechanical function [29].

However, there are currently no universally accepted normal reference values for IOS parameters, and interpretation should be based on laboratory-specific reference ranges adjusted for age, sex, and height. During testing, coughing, breath-holding, or improper posture may lead to transient increases in some parameters, and repeat testing may be required. In addition, severe obesity, which increases chest wall resistance, and conditions such as pleural effusion or pneumothorax, which compress lung tissue, may mask airway functional abnormalities. Therefore, IOS results should be interpreted in combination with chest imaging findings.

This study has several limitations. First, as a single-center retrospective study, selection bias may be present. Because of the retrospective design, information on specific treatment regimens, dosage, treatment duration, and antifibrotic therapy was not consistently available for all patients. Second, long-term follow-up was not performed; therefore, the predictive value of small airway dysfunction for long-term outcomes in patients with RA-ILD, including survival, disease progression, and risk of acute exacerbation, could not be determined. Third, pathological and histological data from the airway mucosa were not available, which limited further clarification of the pathological morphology and molecular mechanisms underlying airway dysfunction in RA-ILD. Fourth, Pearson correlation only reflects linear correlations rather than independent causal or predictive relationships. Performing numerous unadjusted pairwise correlations increases the risk of Type I error, which constitutes a major methodological limitation of this study. Future multicenter prospective cohort studies combining quantitative airway analysis on chest HRCT, pathological assessment, and other techniques are needed to further investigate the mechanisms of airway dysfunction in RA-ILD and to clarify whether small airway function parameters have prognostic or monitoring value.

## 5. Conclusions

In this study, patients with RA-ILD showed evidence of small airway dysfunction, with altered impulse oscillometry parameters suggesting increased airway resistance and reduced airway compliance. Small airway function parameters were associated with FVC% and GAP score, indicating that small airway dysfunction may be related to disease severity in RA-ILD. Assessment of small airway function may provide supplementary information for the clinical evaluation and monitoring of patients with RA-ILD.

## Figures and Tables

**Table 1:** Comparison of baseline characteristics between the RA-ILD group and the control group.

	RA-ILD (255 Cases)	Control (230 Cases)	t Value/χ^2^	*p* Value
Sex (Male/Female)	85:170	79:151	0.06	0.81
Age	67.53 ± 10.13	66.52 ± 9.32	0.93	0.63
Smoking history (%)	25.1%	22.6%	0.41	0.52
Underlying diseases				
Hypertension	124	104	0.57	0.45
Diabetes mellitus	52	30	4.65	0.03
Coronary heart disease	36	13	9.54	0.002
Cerebrovascular disease	10	5	1.23	0.27
Others	15	8	1.55	0.21
BMI (kg/m^2^)	24.39 ± 3.41	26.19 ± 3.52	−4.64	<0.001
FVC%	93.06 ± 16.62	102.85 ± 12.39	−5.79	<0.001
TLC%	95.72 ± 16.49	101.86 ± 12.87	−3.62	<0.001
DLCO%	69.91 ± 20.20	89.76 ± 13.72	−9.86	<0.001

Other comorbidities included reflux esophagitis, atrophic gastritis, gastric ulcer, atrial fibrillation, renal calculi, and other conditions. BMI: Body Mass Index; FVC%: Forced Vital Capacity percentage predicted; TLC%: Total Lung Capacity percentage predicted; DLCO%: Diffusing capacity of the Lung for Carbon Monoxide percentage predicted.

**Table 2:** Comparison of small airway and impulse oscillometry indices between RA-ILD and control group.

	RA-ILD (255 Cases)	Control (230 Cases)	t Value	*p* Value	95%Confidence Interval
MEF25–75 (L/s)	1.96	2.34	−4.3	<0.001	−0.55, −0.21
MEF50 (L/s)	2.91	3.24	−2.65	<0.001	−0.56, −0.08
MEF25 (L/s)	0.91	0.84	−3.48	<0.001	−0.21, −0.06
Z5	0.49 ± 0.19	0.41 ± 0.16	2.99	0.003	0.03, 0.12
R5-R20	0.14 ± 0.16	0.10 ± 0.08	2.28	0.025	0.02, 0.07
R5/R20	1.39 ± 0.26	1.33 ± 0.21	2.06	0.002	0.003, 0.12
X5	−0.20 ± 0.20	−0.14 ± 0.07	−2.32	<0.001	−0.09, −0.03
Fres	17.24 ± 5.80	15.70 ± 3.91	2.51	0.013	0.33, 2.73
MEF25–75%	80.12 ± 33.32	92.57 ± 24.19	−3.69	<0.001	−18.5, −6.4
MEF50%	92.83 ± 40.66	100.02 ± 27.11	−1.99	0.047	−14.28, −0.09
MEF25%	72.36 ± 37.83	81.55 ± 31.69	−2.44	0.015	−16.6, −1.78

**Table 3:** Multiple linear regression analyses evaluating the associations between small airway function parameters and disease severity indicators.

Variable	Dependent Variable	Unstandardized B	95% CI for B	Standardized β	*p* Value
MEF25–75%	GAP score	−0.002	−0.002, −0.001	−0.236	<0.001
MEF50%	GAP score	−0.001	−0.002, −0.001	−0.215	<0.001
MEF25%	GAP score	−0.001	−0.002, −0.000	−0.171	<0.001
MEF25–75%	FVC%	0.194	0.145, 0.242	0.366	<0.001
MEF50%	FVC%	0.144	0.100, 0.187	0.309	<0.001
MEF25%	FVC%	0.095	0.054, 0.137	0.221	<0.001
MEF25–75%	DLCO%	0.109	0.051, 0.167	0.172	<0.001
MEF50%	DLCO%	0.087	0.036, 0.138	0.156	<0.001
MEF25%	DLCO%	0.050	0.002, 0.098	0.097	0.039

## Data Availability

The data presented in this study are available on request from the corresponding author due to regulatory restrictions protecting patient confidentiality and identifiable clinical information.

## Supplementary Materials

The following supporting information can be downloaded at: https://www.mdpi.com/article/10.3390/jcm15156035/s1, Correlation results.
